# Association of cancer history with Alzheimer's disease onset and structural brain changes

**DOI:** 10.3389/fphys.2014.00423

**Published:** 2014-10-31

**Authors:** Kelly N. H. Nudelman, Shannon L. Risacher, John D. West, Brenna C. McDonald, Sujuan Gao, Andrew J. Saykin, for the Alzheimer's Disease Neuroimaging Initiative

**Affiliations:** ^1^Department of Medical and Molecular Genetics, Indiana University School of MedicineIndianapolis, IN, USA; ^2^Training in Research for Behavioral Oncology and Cancer Control, Indiana University School of NursingIndianapolis, IN, USA; ^3^Center for Neuroimaging, Department of Radiology and Imaging Sciences, Indiana University School of MedicineIndianapolis, IN, USA; ^4^Indiana Alzheimer Disease Center, Indiana University School of MedicineIndianapolis, IN, USA; ^5^Indiana University Melvin and Bren Simon Cancer Center, Indiana University School of MedicineIndianapolis, IN, USA; ^6^Department of Biostatistics, Indiana University School of MedicineIndianapolis, IN, USA

**Keywords:** cancer, Alzheimer's disease, inverse association, MRI, gray matter, *APOE*, genetics, ADNI

## Abstract

Epidemiological studies show a reciprocal inverse association between cancer and Alzheimer's disease (AD). The common mechanistic theory for this effect posits that cells have an innate tendency toward apoptotic or survival pathways, translating to increased risk for either neurodegeneration or cancer. However, it has been shown that cancer patients experience cognitive dysfunction pre- and post-treatment as well as alterations in cerebral gray matter density (GMD) on MRI. To further investigate these issues, we analyzed the association between cancer history (CA±) and age of AD onset, and the relationship between GMD and CA± status across diagnostic groups in the Alzheimer's Disease Neuroimaging Initiative (ADNI) cohort study. Data was analyzed from 1609 participants with information on baseline cancer history and AD diagnosis, age of AD onset, and baseline MRI scans. Participants were CA+ (*N* = 503) and CA− (*N* = 1106) diagnosed with AD, mild cognitive impairment (MCI), significant memory concerns (SMC), and cognitively normal older adults. As in previous studies, CA+ was inversely associated with AD at baseline (*P* = 0.025); interestingly, this effect appears to be driven by non-melanoma skin cancer (NMSC), the largest cancer category in this study (*P* = 0.001). CA+ was also associated with later age of AD onset (*P* < 0.001), independent of apolipoprotein E (*APOE*) ε4 allele status, and individuals with two prior cancers had later mean age of AD onset than those with one or no prior cancer (*P* < 0.001), suggesting an additive effect. Voxel-based morphometric analysis of GMD showed CA+ had lower GMD in the right superior frontal gyrus compared to CA− across diagnostic groups (*P*_crit_ < 0.001, uncorrected); this cluster of lower GMD appeared to be driven by history of invasive cancer types, rather than skin cancer. Thus, while cancer history is associated with a measurable delay in AD onset independent of *APOE* ε4, the underlying mechanism does not appear to be cancer-related preservation of GMD.

## Introduction

Multiple epidemiological studies have identified a significant inverse association between cancer and Alzheimer's disease (AD), primarily in white non-Hispanic cohorts (Tirumalasetti et al., 1991; Desouky, 1992; Yamada et al., 1999; Roe et al., 2005, 2010; Driver et al., 2012; Realmuto et al., 2012; Musicco et al., 2013). These studies provide convincing evidence that cancer history reduces the risk of AD in the white non-Hispanic population, with effect sizes ranging from 0.4 to greater than 0.6 (Roe et al., 2005; Driver et al., 2012; Musicco et al., 2013; Roe and Behrens, 2013; Catala-Lopez et al., 2014). Supporting the validity and specificity of this effect, a study by Roe et al. (2010) found the inverse association of cancer specific to AD as compared to vascular dementia. Another study by Musicco et al. (2013) identified the inverse association of cancer and AD in a very large population-based Italian sample accounting for physician and survival bias. This study of invasive cancer types found reduced relative risk of AD in subpopulations of breast, lung, bladder, prostate, and colorectal cancer survivors, though only the colorectal cancer subpopulation risk reduction was statistically significant. Interestingly, the cancers represented in most of these study populations were highly heterogeneous, suggesting that rather than specific cancer effects, such as estrogen deprivation in breast cancer, the inverse association between cancer and AD is likely due to strong underlying biological mechanisms. Identification of these biological mechanisms may provide direction to future therapeutic efforts, particularly for AD, as there is currently a significant lack of effective treatments for this disease.

There are many proposed mechanisms that may explain the inverse association of AD and cancer (Behrens et al., 2009; Holohan et al., 2012; Driver, 2014); a common theory posits that it is primarily driven by genetic predisposition and molecular mechanisms either promoting or suppressing metabolic survival or apoptotic cellular pathways. This metabolic survival theory is supported by a recent paper by Ibanez et al. (2014), that identified genes differentially expressed in AD and several types of cancer concentrated in metabolic and genetic information processing pathways essential for cell survival and apoptotic regulation. As regional neurodegeneration, including loss of gray matter density (GMD), is a hallmark of AD, it was hypothesized that if this theory is correct, older individuals with a history of cancer (CA+) would exhibit preserved GMD compared to those without cancer history (CA−), and that lower GMD in CA− would be related to earlier age of AD onset in contrast to CA+ individuals.

However, cognitive and neuroimaging studies of breast cancer patients provide convincing evidence that CA+ survivors treated with chemotherapy have decreased GMD, more memory concerns, and worse neuropsychological test performance than CA−, up to 20 years post-treatment (Ahles et al., 2002; McDonald et al., 2010; Koppelmans et al., 2012a,b; McDonald et al., 2013; Stouten-Kemperman et al., 2014). There is some evidence to support the negative impact of hormone therapies on perceived and objective cognitive function (Schilder et al., 2009, 2010a; Boele et al., 2014), and that radiotherapy may also be associated with cognitive dysfunction (Shibayama et al., 2014). Furthermore, although the focus of this research to date has been on the effects of cancer treatments on brain structure and function, several studies of breast cancer patients have also found pre-treatment deficits in neuropsychological performance and brain activation, suggesting that CA+ may be associated with cognitive dysfunction, independent of treatment effects (Cimprich et al., 2010; Schilder et al., 2010b; Scherling et al., 2011). Finally, as previously reviewed (Holohan et al., 2013), there have been several imaging studies in heterogeneous cancer populations which have shown differences in brain activation compared to CA−, suggesting that these effects are not limited to breast cancer (Tashiro et al., 1999, 2000, 2001; Golan et al., 2009; Benveniste et al., 2012). It has been suggested based on this evidence that cancer and treatment-related changes may be responsible for an accelerated aging process, particularly in subgroups of more vulnerable patients (Ahles et al., 2012). These results and line of reasoning predict that CA+ should experience greater cognitive dysfunction and neurodegeneration compared to CA−, which may actually worsen over time for some individuals.

This growing body of cancer and cognition literature appears to be in conflict with the metabolic survival theory posited to underlie the inverse association of cancer and AD. To investigate this apparent contradiction, this cohort study utilized the Alzheimer's Disease Neuroimaging Initiative dataset, comprising cognitively normal older adults (CN), participants with significant memory concern (SMC) in the absence of psychometric evidence of cognitive decline, older adults diagnosed with early and late mild cognitive impairment (MCI), and patients with mild clinical AD, to investigate the effect of cancer history on AD-related neurodegeneration. We hypothesized that our findings in this independent sample would be consistent with previous research showing an inverse relationship of cancer and AD. However, also based on previous research, we expected to observe cognitive dysfunction and brain structural changes in cancer patients.

## Materials and methods

### Alzheimer's Disease Neuroimaging Initiative (ADNI)

Data used in the preparation of this article were obtained from the ADNI database (adni.loni.usc.edu). ADNI was launched in 2004 as a collaboration including the National Institute on Aging (NIA), the National Institute of Biomedical Imaging and Bioengineering (NIBIB), the Food and Drug Administration (FDA), pharmaceutical companies, and non-profit organizations. It was framed as a multi-year, public-private partnership, headed by Principal Investigator Michael W. Weiner, MD, VA Medical Center and UCSF. Many co-investigators from over 50 sites across the United States (U.S.) and Canada have contributed to this longitudinal study, recruiting more than 1700 participants (aged 50–90) in three phases, ADNI-1, ADNI-GO, and ADNI-2.

The ADNI study design is described briefly as follows. Participants were collected from across North American in three phases, ADNI-1, ADNI-GO, and ADNI-2; target participant numbers are listed in Table 1. This is not a population study, as the focus was on recruiting participants with specific AD-spectrum diagnoses. ADNI-GO and ADNI-2 added recruitment of early (EMCI) and late MCI (LMCI) to study the full spectrum of AD progression; these participants were all counted as MCI for the purposes of this analysis. As seen in Table 1, while ADNI-1 collected MRI, fluorodeoxyglucose (FDG) positron emission tomography (PET), and Pittsburgh compound B (PiB) PET, later phases of ADNI collected several additional types of neuroimaging data. All data phases collected neuropsychological and self-reported cognitive data, biological samples such as blood for genetic analysis, and demographic and medical history data. Longitudinal protocols included data collection for each participant every 6 months for the first 2 years, and every 12 months after this point. Further information on ADNI study design, protocols, diagnostic criteria, and all measurements utilized in this analysis can be found at http://adni.loni.usc.edu/ and in previous reports (Jack et al., 2010; Jagust et al., 2010; Petersen et al., 2010; Saykin et al., 2010; Trojanowski et al., 2010; Weiner et al., 2010, 2012, 2013). Institutional Review Board approval was obtained by each ADNI site, and informed consent was obtained from each study participant or authorized representative.

**Table 1:** **ADNI study design**.

	Participants*					**Data collection**						
	**CN**	**EMCI**	**MCI**	**LMCI**	**AD**	**MRI**	**fMRI^a^**	**DTI^b^**	**FDG**	**AV45^c^**	**PiB**	**Bios^d^**
ADNI-1	200	–	400	–	200	X			X		X	X
ADNI-GO	↓	200	↓	–	–	X	X	X	X	X		X
ADNI-2	150	150	↓	150	200	X	X	X	X	X		X

a fMRI, functional MRI;

b DTI, diffusion tensor imaging;

c AV45, florbetapir PET amyloid imaging;

d *Bios, biological samples*.

* *Numbers are study targets, not final statistics. Arrows indicate that study participants continued longitudinally in later phases of ADNI*.

### Participants

Self-reported demographic information for all three ADNI phases included baseline age, education, sex, race, ethnicity, and handedness. These factors have all been previously associated with AD diagnosis (Farrer et al., 1997; Fitten et al., 2001; Shadlen et al., 2006; Meng and D'arcy, 2012; Salmon et al., 2013; Yang et al., 2014), and as such were considered potential confounders; participants were excluded from this analysis if they were missing any of this information. Additionally, participants were genotyped for apolipoprotein E (*APOE*) ε2/3/4 alleles as described previously; since *APOE* ε4 is the major known genetic risk factor for late-onset AD and a potential confounder, participants were also excluded if they were missing this data (Saykin et al., 2010; Risacher et al., 2013). All participants included in this analysis met ADNI inclusion and exclusion criteria, which have been described previously, and can be found at http://www.adni-info.org/ (Weiner et al., 2010). A general exclusion rule, as stated in the Procedures Manual, was that a history of any cancer other than non-melanoma skin cancer (NMSC) within 5 years of screening was exclusionary. However, the manual also states that exceptions may be made on a case by case basis. Review of qualitative medical data indicated that there were exceptions made to this rule, primarily for individuals with prostate cancer, but also for individuals with other types of cancer which had been successfully treated and were in remission at the time of study enrollment.

Participants were categorized at baseline as CN, SMC, MCI, or mild AD. More information on measures utilized in diagnosis is available on the ADNI website; basic diagnostic criteria are also briefly summarized as follows. Criteria considered include: subject, informant, and clinician report of memory concerns, memory function documented by neuropsychological testing scores compared to education-adjusted cutoffs on the Logical Memory II subscale (Delayed Paragraph Recall, Paragraph A only) from the Wechsler Memory Scale - Revised (maximum score = 25), Mini-Mental State Exam score out of 30 (Folstein et al., 1975), Clinical Dementia Rating (CDR, range 0–1) (Morris, 1993), and qualitative assessment by a physician of cognitive function and functional performance, guided by the NINCDS/ADRDA criteria (McKhann et al., 1984). CN participants show no signs of depression, memory complaints, MCI, or dementia; neuropsychological memory testing is within the normal range (>8 for 16 or more years of education, >4 for 8–15 years of education, or >2 for 0–7 years of education), they have a Mini-Mental State Exam score between 24 and 30, and have a CDR of 0. SMC individuals exhibit some forgetfulness; however, their informant does not indicate that they are consistently forgetful or experiencing progressive memory impairment. They score within the normal cognitive range for memory function, have MMSEs between 24 and 30, and have a CDR of 0. MCI individuals report subject memory concerns, show abnormal memory function documented by neuropsychological testing (<9 for 16 or more years of education, <5 for 8–15 years of education, or <3 for 0–7 years of education), have MMSEs between 24 and 30, and have a CDR of 0.5; however, their general cognition and functional performance are sufficiently preserved such that a diagnosis of AD cannot be made by the site physician at the time of the visit. Finally, individuals with AD exhibit memory concerns, abnormal memory function documented by neuropsychological testing, have MMSEs between 20 and 26, have CDRs of 0.5 or 1.0, and meet NINCDS/ADRDA criteria for probable AD.

As described in Saykin et al. (2010), *APOE* was genotyped using the two single nucleotide polymorphisms (SNPs) rs429358 and rs7412. A 3 mL sample of blood was taken in ethylenediaminetetraacetic acid (EDTA)-containing vacutainer tubes from all participants. Genomic DNA was extracted at Cogenics (now Beckman Coulter Genomics) utilizing the QIAamp DNA Blood Maxi Kit (Qiagen, Inc., Valencia, CA), following the manufacturer's protocol. Polymerase chain reactions were used to amplify participant DNA, followed by HhaI restriction enzyme digestion, resolution on 4% Metaphor Gel, and visualization by ethidium bromide staining.

Baseline age, education, sex, race, and ethnicity (white non-Hispanic vs. all other reported races/ethnicities), handedness, and *APOE* ε4 status (0 ε4 alleles vs. at least 1 ε4 allele) were all analyzed for significant differences between cancer and AD diagnostic groups using Pearson Chi-Square and ANOVA methods in SPSS 21 (SPSS Statistics 21, IBM Corporation, Somers, NY), to determine whether these potential confounders should be included in further analyses.

Qualitative and quantitative self-reported medical history data was also obtained for all ADNI study participants. For the purposes of this analysis, all qualitative medical history data was manually curated to obtain a complete, more accurate account of each individual's cancer history than was available based on quantitative data. All cancer types were considered for this analysis, including NMSC. Medical information regarding cancer was broken down into pre-baseline cancer history (yes, 1, CA+; no, 0, CA−), as well as a count of prior cancer incidences. Reports of multiple NMSC were only counted as one cancer incidence, given the benign, prevalent nature of this cancer, as well as the lower quality of documentation regarding exact number of incidences. Cancer types were recorded and divided into 14 categories for analysis. Cancer types with only one incidence that did not fit any other categories were categorized as “Other”; notably, there were only seven of these cancer types, showing that the other 13 categories represent the majority of observed cancer. Chi-square analysis of cancer categories by AD diagnostic group was performed to test for potential sample bias. Post-baseline cancer incidents were not utilized in this study due to the small number (43 total), which were distributed evenly between groups (chi square χ^2^ = 4.054, *p* = 0.256).

### MRI acquisition

MRI scans acquisition varied as part of the three ADNI initiatives. ADNI-1 participants' structural MRI baseline scans were acquired using 1.5 Tesla field strength; ADNI-2 and ADNI-GO both utilized 3 Tesla field strength. All available baseline structural MRI scans were downloaded from LONI (http://adni.loni.usc.edu/) for included ADNI participants. Scans were corrected prior to download as previously described (Jack et al., 2008, 2010).

### Comorbidity association analysis

Cancer history (CA+/CA−; prior to baseline) was analyzed for association with baseline AD diagnosis (four groups) using the Chi-Square test. *Post-hoc* analysis was also performed analyzing three types of cancer history, NMSC, prostate cancer, and breast cancer.

Following these results, survival analysis was performed to analyze age of AD onset by cancer history. A time variable was created utilizing age of AD onset for participants diagnosed with AD before or during the study, and age at most current visit for all other study participants. To address potential sources of bias, this time variable was pre-adjusted for the following confounding variables identified in the demographic analysis: sex, education, handedness, race/ethnicity, and *APOE* ε4 allele status. A censor variable was used to denote AD (1) and non-AD (0) participants. Cox regression and Kaplan-Meier survival analyses were conducted utilizing these time and censor variables with CA± status as the factor of interest. Median age of AD onset and 95% confidence intervals (CI) were estimated using the Kaplan-Meier method, and Cox regression forward Wald tests were used to generate Chi-square statistics, significance, and odds ratios (OR), as well as graphical representations. A similar analysis using number of prior cancer incidences as the factor of interest was also conducted. Finally, *post-hoc* analysis investigated the association of the two most common cancer types, NMSC and prostate cancer, with pre-adjusted age of AD onset, using similar methods. Analyses were conducted using SPSS 21.

Of the 1609 included study participants, 257 individuals converted to AD post-baseline, bringing the AD group sizes for this analysis to 160 CA+ AD and 410 CA− AD (total *N* = 570), while CA+ (*N* = 343) and CA− (*N* = 696) included in other diagnostic groups were censored. For the AD cancer history number of incidences analysis, 23 individuals out of the total 570 had a history of two cancers; no individuals with AD at baseline or individuals who converted to AD during the study had more than two prior cancers.

### Image analysis

Scans were processed for voxel-based morphometry (VBM) analyses in Statistical Parametric Mapping 8 (SPM8; Wellcome Department of Cognitive Neuroscience, London, UK), using an updated version of procedures described in previous reports (Ashburner and Friston, 2000; Risacher et al., 2009, 2010, 2013). The majority of participants had at least two scans from the baseline visit; the first acquired scan of acceptable quality was used. Briefly, scans were co-registered to a T1-weighted template, segmented into gray matter, white matter, and CSF compartments with bias correction, and normalized unmodulated to Montreal Neurologic Institute (MNI) space as 1 × 1 × 1 mm voxels. Smoothing was performed with an 8 mm Gaussian kernel. Extensive quality control was performed on all scans. 1609 ADNI participants had all baseline demographic and medical data and baseline scans that passed all quality control measures.

VBM analysis of GMD was performed in SPM8 to analyze differences between AD/cancer groups. The 1609 included participants were divided into eight groups based on AD diagnostic group and CA± status, and baseline corrected scans were analyzed for group differences using a full factorial model, covarying for potential confounding variables including study phase, field strength (1.5 Tesla or 3 Tesla), total intracranial volume (ICV), age, sex, education, handedness, race/ethnicity (white non-Hispanic vs. all else), and *APOE* ε4 allele status. The SPM8 standard gray matter explicit mask was included in the model. Initial results suggested that this mask may not exclude some differences in white matter regions within the brain stem and cerebellum, likely noise caused by atrophy in the AD group; the SPM8 white matter exclusive mask was used to confirm that these changes occurred in white matter.

Weighted contrast vectors were entered for each group in the design matrix to test hypotheses regarding differences in neurodegeneration across the eight AD/cancer groups. AD and MCI groups were expected to show greater neurodegeneration across large regions of the brain compared to other groups; to confirm this, a linear model of less GMD for each group further along the AD spectrum was applied (−2 for AD CA+/CA−, −1 for MCI CA+/CA−, 1 for SMC CA+/CA−, and 2 for CN CA+/CA−). The critical significance voxel-wise threshold (Pcrit) was set to 0.001 uncorrected, and the minimum cluster extent (k) for this contrast was set to 0; given the extensive GMD loss observed in AD and MCI, there was no correction for cluster size included. To test the hypothesis that cancer history was inversely associated with neurodegeneration, the model included weights of +1 for each CA+ group and −1 for each CA− group. CA+/CA− changes within each AD diagnostic group were considered in a similar fashion, and AD CA+/CA− were also contrasted with CN CA+/CA−. For each hypothesis, inverse models were also tested to confirm the specificity of the findings. The critical significance voxel-wise threshold (Pcrit) was set to 0.001 uncorrected, with a minimum cluster extent (k) of *P* ≤ 0.1 uncorrected voxels for these contrasts.

For the significant cluster identified in voxel-wise model of CA+ lower GMD across diagnostic groups, mean cluster GMD value was extracted for all individuals using MarsBar in SPM8 (Brett et al., 2002). These values were analyzed and graphed in SPSS 21 to further investigate diagnostic group differences in GMD change. One outlier from the MCI CA− group with GMD greater than three standard deviations from the mean was excluded. These values were also analyzed with the General Linear Model Univariate ANOVA method, testing for association with types of cancer, covarying for demographic variables previously listed as well as baseline AD diagnostic group. Types of cancer tested included the four largest categories (NMSC, prostate, breast, and melanoma), as well as a category including all cancer types except NMSC. To test specifically whether non-malignant, non-invasive NMSC was associated with this effect, this cancer category was modified for this analysis to exclude individuals who had also had any other type of cancer (57 individuals excluded from 246).

## Results

### Demographic analysis

The 1609 individuals analyzed in this study were obtained as follows. 1818 individuals had ADNI medical history files. Of these, 1780 also had pertinent demographic information (as listed in Table 2). Out of these individuals, 1609 had quality-controlled MRI scans available for analysis.

**Table 2:** **ADNI total cohort demographics (*N* = 1609)**.

	**CA+ CN**	**CA− CN**	**CA+ SMC**	**CA− SMC**	**CA+ MCI**	**CA− MCI**	**CA+ AD**	**CA− AD**	***P*^a^**
ADNI-1	75	135	0	0	108	258	45	133	
ADNI-GO	0	1	0	0	50	78	0	0	
ADNI-2	54	126	34	44	99	234	38	97	
ADNI Total	129	262	34	44	257	570	83	230	<0.001^*^
Age	76 (5.4)	74 (5.9)	73 (6.4)	71 (4.8)	75 (6.9)	72 (7.8)	77 (7.7)	74 (7.8)	<0.001^+^
Education	15 (3.3)	15 (2.9)	16 (2.8)	16 (2.8)	17 (2.5)	17 (2.3)	17 (2.5)	16 (2.7)	<0.001^+^
% Male	68%	48%	68%	55%	53%	48%	61%	45%	<0.001^*^
% R-Hand	97%	89%	88%	87%	88%	91%	92%	95%	0.037^*^
% White, NH	94%	81%	94%	87%	95%	88%	94%	88%	<0.001^*^
% *APOE* ε4+	29%	28%	38%	27%	49%	49%	60%	68%	<0.001^*^
% Ever smoked	49%	35%	59%	50%	39%	40%	45%	36%	0.021^*^

*All included values collected at baseline; participants from ADNI-1 who continued in ADNI-GO or 2 are reported as ADNI-1. Age and education values in years are mean (standard deviation). % R-Hand, % right handed; % White NH, % white non-Hispanic individuals; % APOE ε4+, % individuals with at least one APOE ε4 allele*.

a P-value for Pearson Chi-Square(^*^) or ANOVA(^+^) with dependent variables listed and independent variable for treatment group/cancer history (eight groups, as shown).

Demographic and disease characteristics of the cohort are summarized in Table 2. Age, sex, education, handedness, race/ethnicity, and *APOE* ε4 allele status were all significantly different between AD/cancer groups. There was a significant, expected association between cancer history and smoking (Chi-square χ^2^ = 4.2, *P* = 0.024), but smoking only showed a trend for association with AD diagnostic groups (χ^2^ = 6.8, *P* = 0.078), with a higher portion of SMC individuals reporting they had ever smoked. Since a higher portion of individuals with cancer were also SMC, the trend for smoking association is likely confounded by cancer history. Given this result, smoking was not included as a covariate in subsequent analyses.

**Figure 1:**
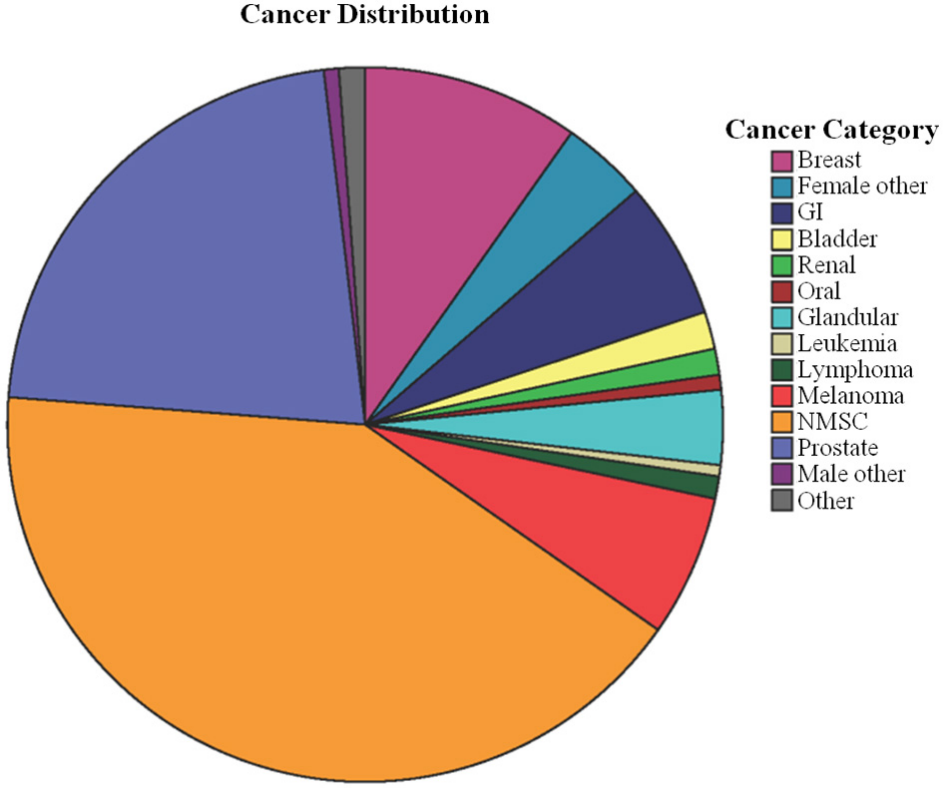
**Categorized cancer types count out of 593 total incidences**. 14 categories were created from the original 40 cancer types; most are self-explanatory, such as all types of cancer related to female organs aside from breast categorized as “Female Other.” The “Other” category contained seven types of cancer with one reported case, which did not fit into any other category. GI, gastrointestinal cancer (including colorectal cancer); NMSC, non-melanoma skin cancer.

Given that previous studies used highly heterogeneous cancer populations, the distribution of cancer types in ADNI was further examined. Out of the 1609 individuals utilized in this analysis, there were 421 individuals with a history of one prior cancer, and 82 individuals with a history of multiple cancers, yielding 593 total recorded cancer incidences. Cancer types were classified into 14 categories, as shown in Figure 1 and Table 3. Although there are some differences in cancer distribution among groups, overall, cancer category percentages were not significantly different between diagnostic groups (Chi-square χ^2^ = 31.2, *P* = 0.8). Subsequent analyses investigating the inverse association of cancer and AD were therefore performed using all types of cancer unless otherwise stated.

**Table 3:** **Cancer categories count and percentage by diagnostic group**.

**Cancer category**	**CN**	**SMC**	**MCI**	**AD**	**Total**
NMSC	74 (46.0%)	19 (48.7%)	123 (40.6%)	30 (33.3%)	246 (41.5%)
Prostate	29 (18.0%)	4 (10.3%)	73 (24.1%)	24 (26.7%)	130 (21.9%)
Breast	19 (11.8%)	5 (12.8%)	25 (8.3%)	9 (10.0%)	58 (9.8%)
Melanoma	10 (6.2%)	5 (12.8%)	16 (5.3%)	7 (7.8%)	38 (6.4%)
GI	9 (5.6%)	2 (5.1%)	22 (7.3%)	4 (4.4%)	37 (6.2%)
Female other	8 (5.0%)	1 (2.6%)	8 (2.6%)	6 (6.7%)	23 (3.9%)
Glandular	3 (1.9%)	2 (5.1%)	12 (4.0%)	3 (3.3%)	20 (3.4%)
Bladder	2 (1.2%)	1 (2.6%)	4 (1.3%)	3 (3.3%)	10 (1.7%)
Renal	1 (0.6%)	0 (0.0%)	4 (1.3%)	2 (2.2%)	7 (1.2%)
Lymphoma	2 (1.2%)	0 (0.0%)	3 (1.0%)	1 (1.1%)	6 (1.0%)
Male other	1 (0.6%)	0 (0.0%)	3 (1.0%)	0 (0.0%)	4 (0.7%)
Oral	1 (0.6%)	0 (0.0%)	3 (1.0%)	0 (0.0%)	4 (0.7%)
Leukemia	1 (0.6%)	0 (0.0%)	1 (0.3%)	1 (1.1%)	5 (0.5%)
Other	1 (0.6%)	0 (0.0%)	6 (2.0%)	0 (0.0%)	7 (1.2%)
Total	161 (100%)	39 (100%)	303 (100%)	90 (100%)	593 (100%)

*Values are expressed as count (percentage of cancer category out of total within each diagnostic group). “Other” category consists of cancer types that do not fit within another category; these included one each of the following cancer types: bone, chondrosarcoma, gallbladder, lung, meningioma, liposarcoma, and a foot tumor of unspecified origin. NMSC, non-melanoma skin cancer; GI, gastrointestinal*.

In addition to baseline data collection, participants were also assessed at a number of follow-up visits, including visits at month(M)6, M12, M18, M24, M36, M48, M60, M72, M84, and M96. Though all other analyses concern data collected at baseline, longitudinal information on participant age and diagnosis at most current (latest) visit was downloaded on July 29, 2014 from the ADNI website (http://adni.loni.usc.edu/) for use in age of AD onset analyses discussed below. The numbers of participants at each most current visit were as follows: baseline (*N* = 115), M6 (*N* = 132), M12 (*N* = 292), M18 (*N* = 25), M24 (*N* = 533), M36 (*N* = 223), M48 (*N* = 49), M60 (*N* = 26), M72 (*N* = 52), M84 (*N* = 101), and M96 (*N* = 61). Because ADNI-GO and ADNI-2 are newer initiatives, participants in these phases of the study do not yet have visits beyond M48; data collection is ongoing. Of the 115 individuals with no visits beyond baseline, most participants withdrew voluntarily after this visit, for reasons including scheduling, discomfort, or unwillingness to comply with protocols (particularly lumbar puncture), or partner/caregiver burden. There were 12 participants for whom there was no available data on reason for loss to follow-up, and an additional five participants who could not be contacted after the initial visit. Among the remaining participants with baseline data, there were four participant deaths (three AD and one MCI), and seven participants who withdrew due to stated medical issues (two CN, four MCI, and one AD). Given the small number of participant withdrawals attributable to medical issues and death, it is unlikely that this is a source of bias for the longitudinal data analysis.

### AD and cancer inverse association

**Figure 2:**
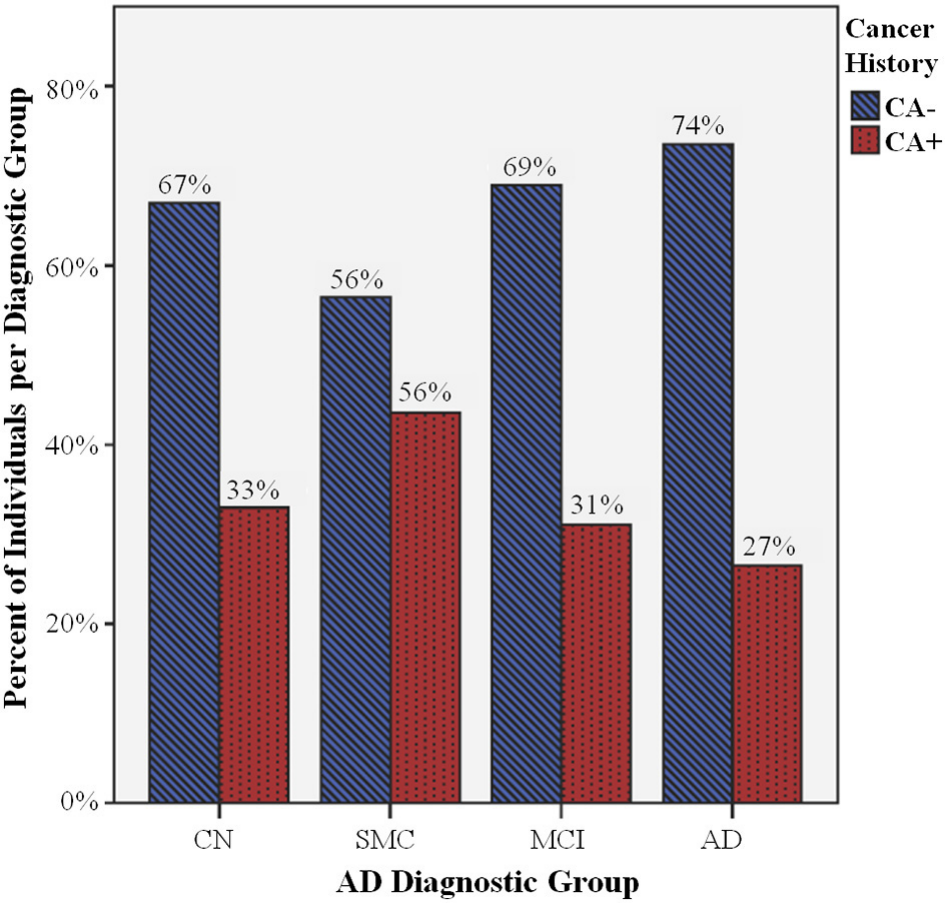
**Percent of individuals with cancer history (CA+) per diagnostic group**. There are significant differences in CA+ (blue striped bars) compared to individuals without cancer history (CA−, red dotted bars) between Alzheimer's disease (AD) diagnostic groups (*P* = 0.025), including cognitively normal controls (CN), and individuals with significant memory concerns (SMC), mild cognitive impairment (MCI), and AD. There is a smaller percentage of AD CA+, and a larger percentage of SMC CA+, compared to the CN CA+ percentage, while the MCI CA+/CA− ratio does not appear to be significantly different than CN CA+/CA−.

Chi-Square analysis indicated that CA+ was significantly associated with AD diagnostic group at baseline (χ^2^ = 9.4, *P* = 0.025). As seen in Figure 2 and Table 4, fewer study participants with AD are CA+ compared to other diagnostic groups. Interestingly, individuals with SMC are more evenly divided between CA+ and CA− than other groups.

**Table 4:** **Cancer history by baseline AD diagnostic group**.

	**All cancer types**		**NMSC**		**Prostate cancer**		**Breast cancer**	
	**CA+**	**CA−**	**CA+**	**CA−**	**CA+**	**CA−**	**CA+**	**CA−**
CN	129 (33%)	262 (67%)	74 (19%)	317 (81%)	29 (7%)	362 (93%)	19 (5%)	372 (95%)
SMC	34 (44%)	44 (56%)	19 (24%)	59 (76%)	4 (5%)	74 (95%)	5 (6%)	73 (94%)
MCI	257 (31%)	570 (69%)	123 (15%)	704 (85%)	73 (9%)	754 (91%)	25 (3%)	802 (97%)
AD	83 (26%)	230 (74%)	30 (10%)	283 (90%)	24 (8%)	289 (92.3%)	9 (3%)	304 (97%)
*P*^a^	0.025		0.001		0.610		0.190	

*Values are expressed as count (percentage within diagnostic group). NMSC, non-melanoma skin cancer*.

a *P-values for Chi-square analyses of each listed cancer type (or all) by diagnostic group*.

*Post-hoc* analysis examined the largest cancer category, NMSC, for association with AD diagnostic group. Chi-square analysis indicated that there were significantly fewer individuals with a history of NMSC in the AD diagnostic group (10%) compared to 15% or greater for all other diagnostic groups (χ^2^ = 16.9, *P* = 0.001; Table 4), supporting inclusion of this cancer type in analyses. Interestingly, no such trend was observed for prostate cancer (χ^2^ = 1.8, *P* = 0.61; Table 4). Breast cancer showed a trend for fewer individuals in the AD and MCI groups compared to SMC and CN, but this trend did not reach statistical significance (χ^2^ = 4.8, *P* = 0.19; Table 4). Other cancer types were not examined due to insufficient power.

### Survival analysis of age of AD onset

**Figure 3:**
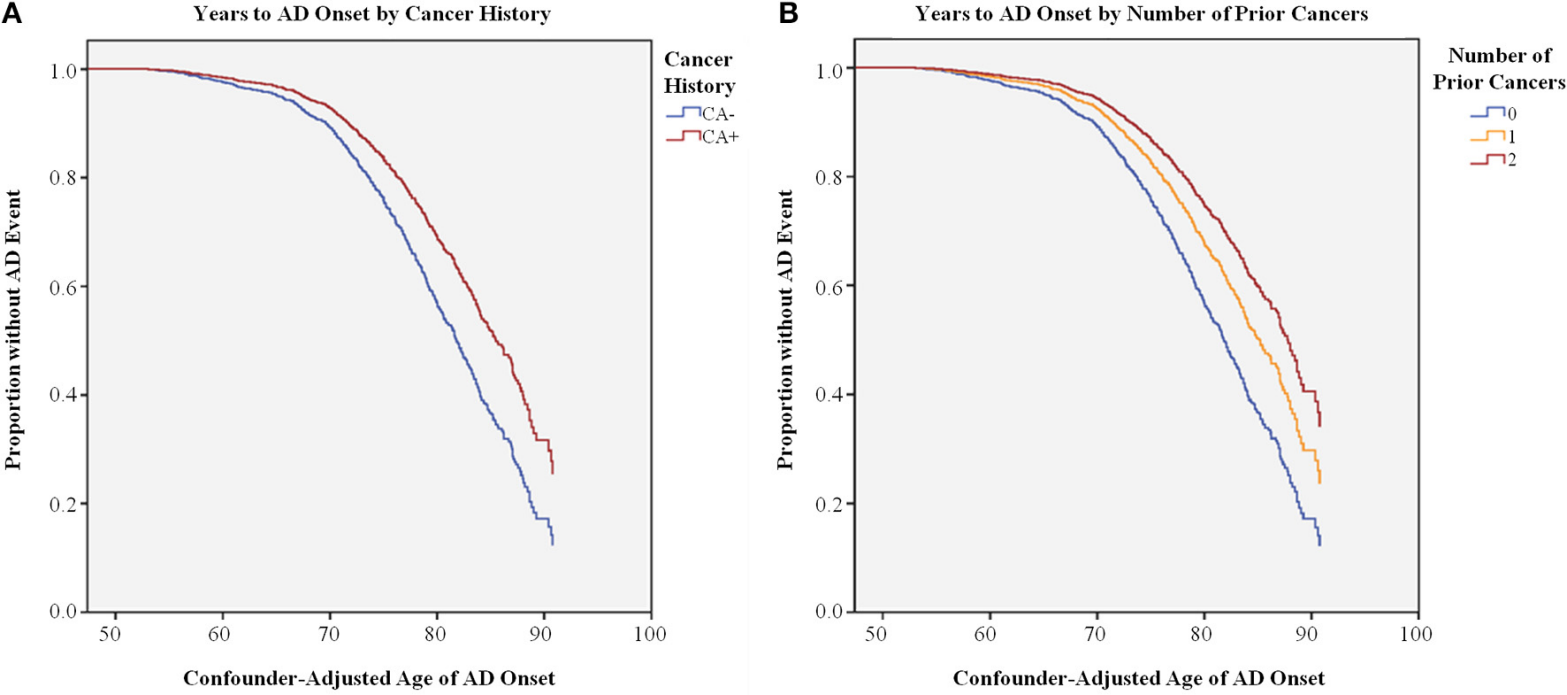
**Survival Analysis of Age of AD onset by Cancer History**. **(A)** Cox regression of confounder-adjusted age (years) of AD onset for individuals with (CA+, red line) or without (CA−, blue line) cancer history, indicating that CA+ have later age of AD onset compared to CA− (*P* < 0.001). **(B)** Cox regression of confounder-adjusted age (years) of AD onset for individuals with 2 (dark red line), 1 (orange line), or 0 (blue line) prior cancer incidences, indicating that individuals with 2 prior cancer incidences have later age of AD onset compared to individuals with 1 or 0 incidences (*P* < 0.001).

Kaplan-Meier survival analysis of age of AD onset with cancer history indicated that those with CA− history had significantly earlier median age of AD onset, as seen in Figure 3A and Table 5; Cox regression shows that cancer history is protective against AD, with CA− 1.5 times more likely to develop AD compared to CA+ (P < 0.001). Importantly, because this analysis was adjusted for *APOE* ε4, these results also suggest that cancer history-associated later age of AD onset is independent of this risk factor. Furthermore, this effect appears to be additive, as CA+ with one prior cancer are still 1.3 times more likely to develop AD compared to CA+ with two prior cancers (*P* < 0.001; Figure 3B, Table 5).

**Table 5:** **Age of AD onset (AoO) by cancer history**.

**Method**	**Kaplan-Meier**		**Cox regression**			
**Measure**	**Median AoO**	**95% CI**	**χ^2^**	**P**	**AD OR**	
All: CA−	81.7	80.7–82.8	20.9	0.000	1.5	1.3–1.8
All: CA+	84.7	83.4–86.0			^*^Ref	
All: 0 CA−	81.7	80.7–82.8	22.2	0.000	2.0	1.3–3.0
All: 1 CA+	84.3	83.2–85.4			1.3	0.9–2.1
All: 2 CA+	85.7	82.4–88.9			Ref	
NMSC: CA−	82.4	81.5–83.3	13.3	0.000	1.6	1.2–2.1
NMSC: CA+	85.7	82.7–88.6			^*^Ref	
Prostate: CA−	82.8	82.0–83.7	4.4	0.037	1.4	1.0–1.8
Prostate: CA+	84.7	82.6–86.9			Ref	

Values for Median AoO and 95% CI are given in years. AoO, age of onset; CI, confidence interval; AD OR, odds ratio of developing Alzheimer's disease;

* *Ref, reference variable for odds ratio calculation*.

*Post-hoc* analysis of the two largest cancer categories indicated that CA+ NMSC showed the cancer protective effect against AD; CA− were 1.6 times more likely to develop AD compared to individuals with a history of NMSC (*P* < 0.001; Table 5). A similar protective effect was also observed for prostate cancer, though this effect was not as significant (*P* = 0.037; Table 5), possibly due to the smaller number of individuals with this cancer (see Table 3).

### GMD differences between groups

**Figure 4:**
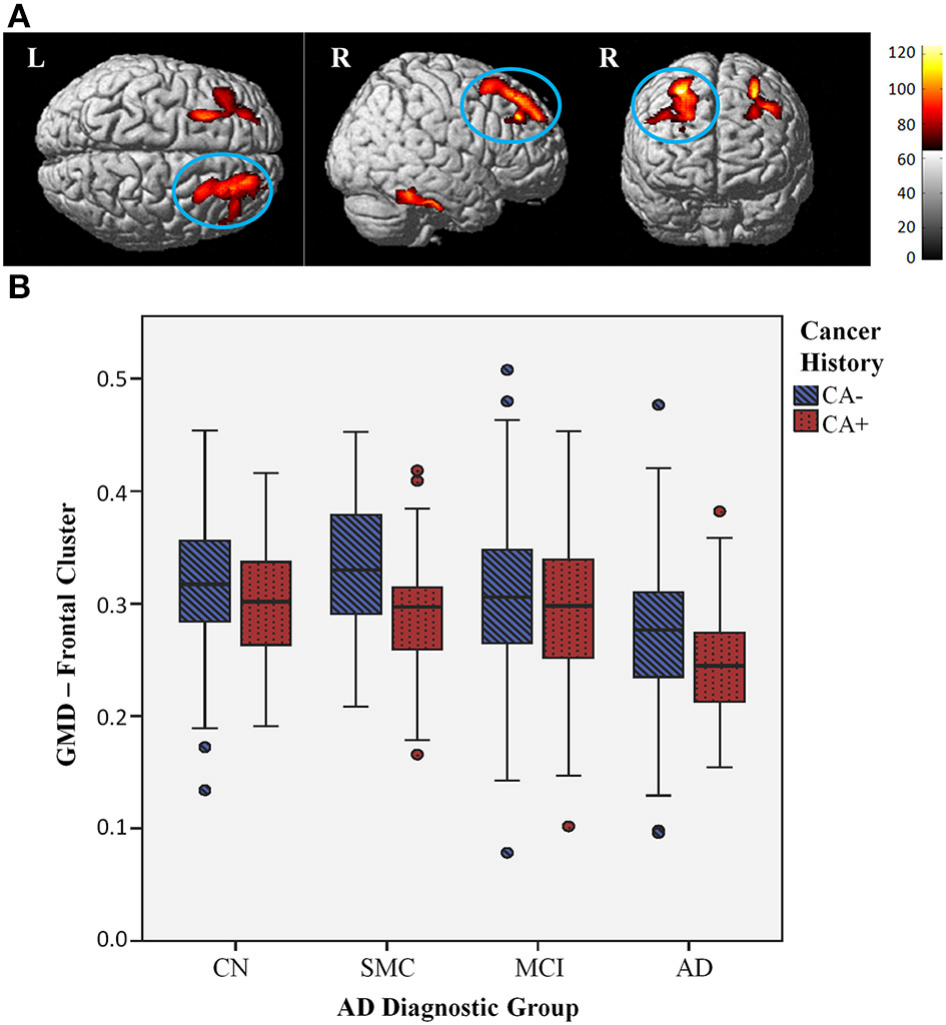
**Lower gray matter density (GMD) with cancer history across diagnostic groups**. **(A)** Surface rendering shows individuals with a history of cancer (CA+) display lower GMD than individuals without cancer history (CA−), across diagnostic groups, in the right superior frontal gyrus (cluster maximum MNI coordinates 28, 32, 54), shown circled (*P*_unc_ < 0.001, cluster threshold *P*_unc_ ≤ 0.1); this effect is observed to be bilateral at a more lenient threshold (*P*_unc_ < 0.01, cluster threshold *P*_unc_ ≤ 0.1), shown above. Colored areas indicate regions where CA+ gray matter density was less than CA− across groups at this threshold; red to yellow color scale indicates increasing statistical significance, with yellow areas indicating the most significant regions. **(B)** GMD values for right superior frontal gyrus cluster graphed by CA+ (red dotted bars) and CA− (blue striped bars) across AD diagnostic groups; CA+ have lower GMD across diagnostic groups.

As noted above, a linear model of GMD deficits in AD, MCI, and SMC groups compared to CN was used to confirm that groups further along the AD spectrum display lower GMD. As expected, lower GMD was observed for affected groups throughout the brain, consistent with prior work (Risacher et al., 2009, 2010). Modeling the opposite relationship, with higher GMD in the AD group compared to other groups, showed no significant regions of greater GMD in the AD group. A second VBM analysis examined all groups irrespective of AD diagnosis, to identify regions that were increased or decreased in CA+ compared to CA−. This model showed that CA+ had lower GMD in the right superior frontal gyrus compared to CA− (peak-level *P*_unc_ < 0.001, cluster-level *P*_unc_ ≤ 0.1; Figure 4A). There were no regions of significantly greater GMD in CA+ at this threshold. As seen in Figure 4B, CA+ showed significantly lower GMD in the right superior frontal gyrus cluster across diagnostic groups.

This finding did not appear to be influenced by disease progression, as AD individuals did not display any significant differences compared to CN. Furthermore, comparing CA+ vs. CA− within each group did not yield any significant regions at this threshold. A lack of significant cortical and subcortical GMD differences between CA+/CA− within groups suggests that the lower CA+ frontal GMD is not being driven by any particular group, but rather is an underlying difference common to all CA+ in this study cohort.

To further investigate this finding, GMD cluster values were tested for association with different types of cancer, controlling for AD diagnostic group and demographic variables. As expected, GMD was significantly associated with all cancer types (*F* = 10.0, *P* = 0.002), which was still significant after excluding individuals with NMSC (*F* = 4.9, *P* = 0.027). GMD was associated with prostate cancer (*F* = 4.3, *P* = 0.039), and showed a trend for association with breast cancer (*F* = 3.7, *P* = 0.055). Interestingly, GMD only showed a trend for association with NMSC (*F* = 3.1, *P* = 0.081), and showed no association with melanoma (*F* = 0.0, *P* = 0.916).

## Discussion

These findings show a significant inverse association between cancer and subsequent development of AD in the ADNI cohort, in concordance with previous epidemiological studies. Importantly, while previous studies have indicated that this inverse association is mediated by age, our results are the first to quantify the later age of AD onset associated with cancer history, as well as to suggest that this effect may be additive, as the small group of individuals with a history of multiple cancers showed later age of AD onset compared to individuals with a history of one or no cancers. Furthermore, these data demonstrated that NMSC, which has not been included in most other studies, was a significant driver of this effect. This suggests that the malignancy of the cancer may not be an important factor driving the inverse association with AD. Alternatively, it may highlight potential environmental mechanisms, such as sun exposure and subsequent increase in vitamin D.

In order to obtain a more complete context for this analysis, cancer history data was compared to U.S. population-level cancer data using the SEER Cancer Statistics Review, 1975–2011 data for cancer incidence and 36-year limited duration prevalence. SEER data indicates that breast and prostate cancer are the most common cancer types (with very similar incidence and prevalence), followed by colorectal cancer and melanoma. However, this report did not include NMSC, which would be expected to have a higher incidence and prevalence (given that squamous and basal cell carcinomas are largely benign, non-invasive cancer types), as observed in the ADNI cancer history data. In ADNI, history of prostate cancer is more common than breast cancer, contrary to SEER incidence and first cancer prevalence. This is perhaps not surprising in this context, as prostate cancer has a later onset and thus may have a higher prevalence as a second or third cancer than other cancer types. Additionally, there may be more prostate cancer in the ADNI sample because there are more males with cancer participating in this study than females (65% male CA+). Accounting for these demographic differences, the cancer history data in the ADNI cohort appears to be relatively similar to national incidence and prevalence estimates.

There was one notable exception to the correspondence of ADNI data with the SEER national incidence data. Interestingly, there was only one reported case of an individual with a prior incidence of lung cancer in the 1609 individuals included in this study. This may be due to survival bias; although lung cancer incidence is quite common (comparable to SEER prostate cancer age-adjusted incidence in white individuals), lung cancer patients have very low SEER 5-year survival compared to other types of cancer (less than 20% for white men and women) (Howlader et al., 2014).

There are a few other limitations of this study that are important to consider. Across diagnostic groups, CA+ individuals were, on average, older than CA−. This may represent an inherent study bias; given that CA+ individuals have a later age of AD onset, older CA+ than CA− would be expected in the AD group. However, it is interesting that this trend was also observed in other diagnostic groups, including CN. One possible explanation may be that in addition to later age of AD onset, CA+ individuals also experience later onset of age- or neurodegeneration-related memory concerns, which may be a motivator to enroll in this type of study. While this is an important caveat to keep in mind when interpreting the current results, the Kaplan-Meier survival analysis method, used to examine age of AD onset with all study participants, was chosen to minimize this limitation, and as noted above all neuroimaging analyses covaried for age. There were several other demographic differences between CA+/CA− groups; CA+ had a higher percentage of white non-Hispanic individuals, likely due to the inclusion of all types of skin cancer in this category, and CA+ also had more males, likely due to the prevalence of prostate cancer in older men, as observed here. Again, all neuroimaging analyses covaried for these demographic confounds. The potential contribution of *APOE* ε4 to cancer was also considered; though *APOE* ε4 alleles are significantly different between diagnostic groups as noted in the demographics table, individuals with *APOE* ε4 alleles were not more likely to have cancer history than those without at least one *APOE* ε4 allele, and this factor was also covaried for in all neuroimaging analyses to account for its impact on neurodegenerative processes in AD. Age of AD onset analysis stratifying for *APOE* ε4 still found CA+ associated with later age of AD onset, supporting the assertion that *APOE* ε4 does not appear to be driving the inverse association of cancer and AD.

The observed lower GMD in CA+ compared to CA− is not predicted by the common theory of the inverse association of cancer and AD, which suggests that CA− would have lower GMD. However, this finding does fit with studies in cancer patients, which have found gray matter reductions in patients undergoing treatment as well as long-term survivors up to 20 years post-treatment (McDonald et al., 2010, 2013; Koppelmans et al., 2012b). Additionally, in the present study an increased percentage of CA+ was observed in the SMC group compared to other groups, suggesting that while cancer may delay age of AD onset, CA+ individuals may have increased cognitive concerns, consistent with the cancer and cognition literature (Ahles and Saykin, 2007; Mehnert et al., 2007; Weis et al., 2009; Mandelblatt et al., 2013; McDonald et al., 2013). Interestingly, the frontal pattern of lower CA+ GMD occurs in regions similar to those reported in neuroimaging studies of breast cancer and chemotherapy-associated gray matter changes (McDonald et al., 2010, 2013). Sixty-three percent of cancers reported in ADNI were either NMSC or prostate; chemotherapy is not a common treatment for either of these, and chemotherapy is not administered for all patients afflicted with other types of cancer reported in this study, including breast cancer, the next most common cancer type. Therefore, while comprehensive treatment data were not available for these patients, it is probable that the majority did not receive chemotherapy. The CA+ effect observed here could be a synergistic result of cancer-specific changes in addition to a subgroup of patients experiencing chemotherapy treatment-related gray matter effects. The finding that lower GMD is significantly associated with invasive cancer types, and only showed a trend for association with NMSC, further supports this idea. These results highlight the need for more long-term studies of cancer and treatment effects on neuroimaging measures and cognitive dysfunction, particularly in older patients where these medical factors may predispose to neurodegeneration or pose an additional risk for cognitive dysfunction.

Given the finding of lower GMD in CA+, which would be predicted in the context of cancer and cognition literature but is unexpected in the context of cancer and neurodegenerative disease literature, we posit that the inverse association of cancer and AD is more complex than the metabolic survival theory would suggest. As reviewed by Holohan et al. (2012), there are many potential pathways driving this effect, which may have synergistic interactions as well. Considering this information, we propose several alternate biological mechanisms and highlight important directions for future research of this effect. First, while baseline imaging indicates lower GMD in CA+ patients, potentially as a long-term result of cancer and related treatments, this study does not capture the rate at which gray matter is changing over time in these patients. Examining this data will be an important step to confirm the neurodegenerative profile of CA+ compared to CA−. Second, analyzing the impact of cancer history on the amyloid pathway and associated biomarkers may demonstrate an alternate mechanism through which cancer could protect against AD. Given that high levels of inflammatory markers have been associated with poorer survival in cancer, cancer survivors may be selected for lower cytokine genetic load or expression, which may be protective against neurotoxic inflammation pathways linked to amyloid plaque accumulation in AD when compared to unselected individuals with no cancer history. It is also possible that some polymorphisms in the amyloid pathway may be inversely associated with cancer and AD; peptidylprolyl cis/trans isomerase, NIMA-interacting 1 (*PIN1*) has been proposed as one such candidate (Balastik et al., 2007; Driver and Lu, 2010). Finally, a common theory in the cancer and cognition literature posits that cancer patients and survivors have gray matter reductions and significantly increased subjective cognitive concerns, which are not well-correlated with objective neuropsychological performance, potentially due to compensatory activation, wherein the brain recruits additional resources to deal with cognitive challenges. In the ADNI data there was a higher portion of cancer survivors in the SMC group, and cancer survivors had lower GMD than CA−; it is possible that these individuals experience compensatory activation, similar to that previously shown in a functional MRI study of breast cancer patients, which may delay cognitive performance decline and AD diagnosis (McDonald et al., 2012). There are a multitude of pathways which have been implicated in AD and cancer, as discussed in Holohan et al. (2012), which require further functional investigation as well. Future research should investigate other biomarkers of AD, including longitudinal gray matter change, amyloid pathway-specific markers, and inflammatory markers, as well as measures of brain activation in cancer and AD diagnostic groups, to further elucidate the biological mechanisms underlying the inverse association of cancer and AD, with the goal of identifying preventative and therapeutic targets.

### Conflict of interest statement

The authors declare that the research was conducted in the absence of any commercial or financial relationships that could be construed as a potential conflict of interest.
